# The Development of Nanomaterials in Adsorption, Separation and Purification

**DOI:** 10.3390/nano14201660

**Published:** 2024-10-16

**Authors:** Daniela Cristina Culita

**Affiliations:** Ilie Murgulescu Institute of Physical Chemistry, Romanian Academy, 202 Splaiul Independentei, 060021 Bucharest, Romania; dculita@icf.ro

## 1. Introduction

In an era marked by growing concerns about environmental pollution, resource scarcity, and energy demand, nanomaterials are emerging as powerful tools for addressing challenges in water treatment, air purification, and industrial separations. Nanomaterials, due to their unique size-dependent properties and surface chemistry, high surface area, and tunable pore structures, offer innovative solutions for improving the efficiency, selectivity, and sustainability of these processes. Their versatility enables them to capture a wide range of contaminants, including heavy metals, organic pollutants, and toxic gases, which are increasingly contributing to global environmental concerns. The development of nanomaterials tailored for adsorption, separation, and purification has not only improved process efficiency but also reduced energy consumption and waste production, aligning with global goals of sustainability and environmental protection.

## 2. An Overview of Published Articles

The present Special Issue presents recent advancements in the development of nanomaterials for adsorption, separation, and purification processes through six original research articles.

Waheed et al. [1] reported a new method for fabricating Mg/Al LDHs with different Ni concentrations (20% and 30%) and converted it into Mg/Al LDOs by calcination at 400 °C. The behavior of methyl orange (MO) adsorption onto the synthesized samples was assessed using batch adsorption experiments. The authors attributed the adsorption performance of MO to the feeding ratio of Ni^2+^ in NMA-LDOs, as the insertion of nickel ions into the LDH lattice results in additional surface defects and positively charged sites, which increase MO’s chemical affinity.

Pirvu et al. [2] evaluated two adsorbent nanomaterials, Fe_3_O_4_ and ZSM-5 zeolite, for the removal of acetaminophen from wastewater. Fe_3_O_4_ showed the highest adsorption capacity (68.9 mg/g) and removal efficiency (84.6%) at a pH of 6, while ZSM-5 achieved an adsorption capacity of 49.5 mg/g and a removal efficiency of 75%. The difference in performance was attributed to surface area, porosity, and preparation methods.

Sewnet et al. [3] reported a graphitic carbon nitride (g-C_3_N_4_) photocatalyst developed using a single-step calcination of urea and thiourea mixtures at various temperatures. They evaluated the impact of calcination temperatures on the photocatalytic activity of g-C_3_N_4_ in degrading Rhodamine B (RhB) under visible LED light, with optimal performance achieved at 600 °C. The authors concluded that this temperature facilitated the efficient separation of photoinduced charge carriers, high crystallinity, and enhanced visible light absorption range, resulting in superior photocatalytic activity.

Musat et al. [4] developed ternary core-hybrid shell structures of FexOy@SiO_2_/ZnO combining the co-precipitation of iron oxide cores (FexOy) with an ultrasonic-assisted sol–gel method using commercial ZnO nanoparticles. These structures were then decorated with silver nanoparticles (Ag NPs). The photocatalytic and antimicrobial activities of the resulting nanomaterials were evaluated and compared to those of the ternary system without silver. The Ag-decorated core-hybrid shell structures demonstrated enhanced adsorption and photocatalytic activity for methylene blue, likely due to the presence of Ag nanoparticles, reduced exciton recombination, along with the contributions of ZnO in the hybrid shell, and an increased specific surface area. Additionally, the Ag-decorated core-hybrid shell Fe_3_O_4_ exhibited significant antimicrobial activity against *S. aureus* and effectively inhibited the growth of Gram-negative strains such as *Ps. aeruginosa* and *E. coli*, as well as a good antifungal activity against *Candida*.

Kuncser et al. [5] synthesized a series of hematite-based nanocomposites (Fe-hbnc, Mn-hbnc, Co-hbnc, Ni-hbnc, Cu-hbnc, and Zn-hbnc) through the controlled thermal decomposition of goethite-based materials. They conducted catalytic tests for the oxidative degradation of indigo carmine dye in simulated wastewater using hydrogen peroxide and concluded that Cu-hbnc exhibited the highest activity. The performance of Cu-hbnc was attributed to its textural properties, specifically pore dimensions that facilitate dye penetration, as well as the basic character of CuO. Compared to other catalytic systems reported in the literature for indigo carmine removal, Cu-hbnc stands out for its high activity under mild conditions (atmospheric pressure and room temperature), eliminating the need for a quartz photoreactor or UV light source as required by other catalysts. Additionally, the catalytic activity of Cu-hbnc was unaffected by the presence of H_2_O_2_.

Simonescu et al. [6] developed three novel magnetic nanocomposites incorporating carboxyl-functionalized SBA-15 silica and magnetite nanoparticles using a convenient and effective method. They evaluated the effectiveness of these nanocomposites as adsorbents for cationic dyes, methylene blue (MB) and malachite green G (MG), from single and binary aqueous solutions. It was found that an increased amount of carboxyl groups on the adsorbent surface enhanced the adsorption capacity for both dyes. Kinetic studies indicated that the dye adsorption process followed a pseudo-second-order kinetics model, with electrostatic interactions identified as the primary adsorption mechanism. The maximum adsorption capacity (256.09 mg g^−1^ for MB and 126.55 mg g^−1^ for MG) for the nanocomposite with the highest amount of carboxyl groups surpassed values reported in the literature for similar materials.

## 3. Conclusions

The development of nanomaterials for adsorption, separation, and purification processes marks a significant advance in addressing contemporary environmental challenges. Their unique properties, such as high specific surface area, enhanced reactivity, and tunable structures, have enabled more efficient removal of pollutants from various matrices, including water, air, and industrial effluents. The promising results from the studies included in this Special Issue demonstrate that nanomaterials can surpass traditional methods in terms of efficiency. Furthermore, the capacity to regenerate these materials for multiple cycles of use increases their economic viability and aligns with the principles of a circular economy. Looking to the future, interdisciplinary research will be essential to optimize the nanomaterials used for the development of adsorption, separation, and purification technologies. Addressing challenges such as scalability, regulatory compliance, and long-term stability will be crucial to the successful integration of nanomaterials into existing systems.
